# Propofol-based total intravenous anesthesia is associated with better survival than desflurane anesthesia in glioblastoma surgery

**DOI:** 10.1371/journal.pone.0255627

**Published:** 2021-08-05

**Authors:** Yi-Hsuan Huang, Zhi-Fu Wu, Meei-Shyuan Lee, Yu-Sheng Lou, Ke-Li Wu, Kuang-I Cheng, Hou-Chuan Lai

**Affiliations:** 1 Department of Anesthesiology, Tri-Service General Hospital and National Defense Medical Center, Taipei, Taiwan, Republic of China; 2 Department of Anesthesiology, Kaohsiung Medical University Hospital, Kaohsiung Medical University, Kaohsiung, Taiwan, Republic of China; 3 Department of Anesthesiology, Faculty of Medicine, College of Medicine, Kaohsiung Medical University, Kaohsiung, Taiwan, Republic of China; 4 School of Public Health, National Defense Medical Center, Taipei, Taiwan, Republic of China; 5 Graduate Institutes of Life Sciences, National Defense Medical Center, Taipei, Taiwan, Republic of China; 6 Postgraduate Year of Medicine Residency Training, Tri-Service General Hospital and National Defense Medical Center, Taipei, Taiwan, Republic of China; Ospedale Sant’Antonio, ITALY

## Abstract

**Background:**

Previous research has shown that anesthetic techniques can influence patient outcomes following cancer surgery. However, the effects of anesthesia in patients undergoing glioblastoma surgery are still not known. We studied the relationship between the type of anesthesia and patient outcomes following elective glioblastoma surgery.

**Methods:**

This was a retrospective cohort study of patients who underwent elective glioblastoma surgery between January 2008 and December 2018. Patients were grouped according to the anesthesia they received, desflurane or propofol. A Kaplan-Meier analysis was conducted, and survival curves were presented from the date of surgery to death. Univariable and multivariable Cox regression models were used to compare hazard ratios for death after propensity matching.

**Results:**

A total of 50 patients (45 deaths, 90.0%) under desflurane anesthesia and 53 patients (38 deaths, 72.0%) under propofol anesthesia were included. Thirty-eight patients remained in each group after propensity matching. Propofol anesthesia was associated with improved survival (hazard ratio, 0.51; 95% confidence interval, 0.30–0.85; *P* = 0.011) in a matched analysis. Furthermore, patients under propofol anesthesia exhibited less postoperative recurrence than those under desflurane anesthesia (hazard ratio, 0.60; 95% confidence interval, 0.37–0.98; *P* = 0.040) in a matched analysis.

**Conclusions:**

In this limited sample size, we observed that propofol anesthesia was associated with improved survival and less postoperative recurrence in glioblastoma surgery than desflurane anesthesia. Further investigations are needed to examine the influence of propofol anesthesia on patient outcomes following glioblastoma surgery.

## Introduction

Glioblastoma (GBM, World Health Organization grade IV) is the most common malignant primary brain tumor, with an incidence of 3.19 cases per 100,000 person-years [1]. GBM is a devastating brain tumor, with only 1 in 4 patients alive at 2 years and a 5-year survival rate of about 5%. Postoperative recurrence is nearly universal despite advances in surgery, radiation, and chemotherapy. Although surgical resection plays an important role in the treatment of GBM [2], surgical intervention may result in neuroendocrine and metabolic changes, which may impair cell-mediated immunity and activate the implantation of circulating tumor cells [3]. This potential combination of impaired immune responses and cancer cell seeding enhances the susceptibility of patients undergoing cancer surgery to the development of postoperative metastasis associated with poor survival. The potential role of anesthetic techniques in cancer survival, postoperative recurrence, or metastasis formation has attracted attention.

Data from human cancer cell lines and animal research showed that different anesthetics might affect the immune system in different paths [4–9]. Research has shown that inhalation anesthesia (INHA) is pro-inflammatory and may affect immune processes, thus increasing the incidence of postoperative metastasis [8–12]. However, propofol seemed to reduce tumor growth and decrease the risk of metastasis in humans and mice [6, 11–14].

Grau et al. [15] showed that propofol anesthesia had no impact on patient survival when compared to INHA (isoflurane, desflurane, or sevoflurane) in GBM surgery. Schmoch et al. [16] reported that propofol anesthesia had no influence on the survival of GBM patients compared with sevoflurane. However, Dong et al. [17] found that propofol may be beneficial in high-grade glioma (World Health Organization grade III and IV) patients with poor preoperative Karnofsky performance status compared with sevoflurane. To date, few studies have compared the effects of desflurane versus propofol anesthesia on patient outcomes following GBM surgery. We hypothesized that patients under desflurane anesthesia might have subsequent poor outcomes than patients under propofol anesthesia, as in our previous cancer studies [18–23]. Thus, we performed a retrospective cohort study to examine whether the choice of anesthetic, desflurane versus propofol, is associated with patient survival and postoperative recurrence following GBM surgery.

## Materials and methods

**Fig 1:**
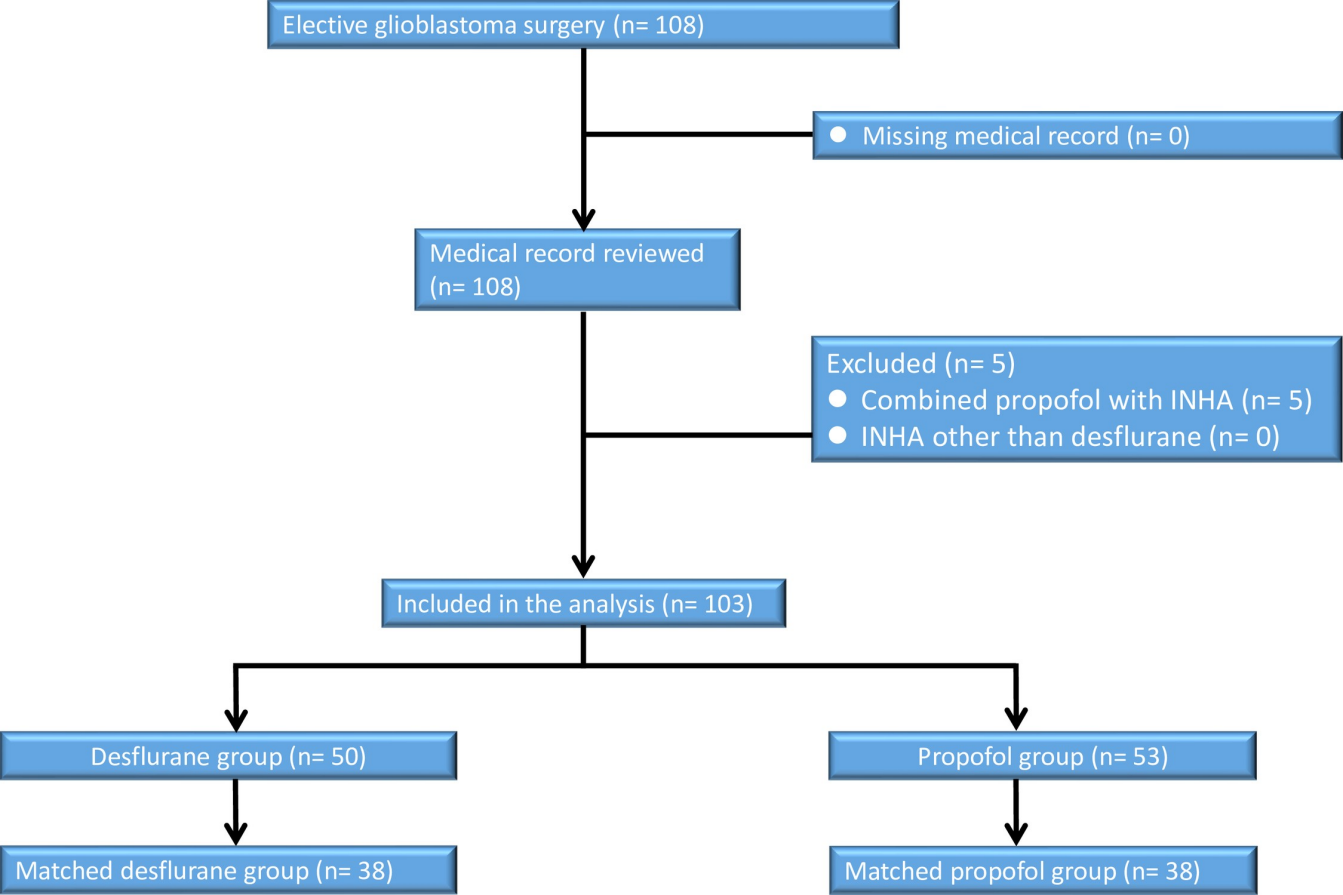
Flow diagram detailing the selection of patients included in the retrospective analysis. 5 patients were excluded due to combined propofol anesthesia with inhalation anesthesia (INHA), incomplete data, or age < 20 years.

This study was conducted at the Tri-Service General Hospital (TSGH), Taipei, Taiwan, Republic of China. The ethics committee of the TSGH approved this retrospective cohort study and waived the need for informed consent (TSGHIRB No: 2-108-05-168). The data was gathered from the electronic database and medical records of the TSGH. From January 2008 to December 2018, 103 consecutive patients with an American Society of Anesthesiologists (ASA) score of II–III who underwent elective primary GBM surgery with propofol anesthesia (n = 53) or desflurane anesthesia (n = 50) were eligible for analysis. The type of anesthesia was chosen according to the anesthesiologist’s personal preference. The exclusion criteria were propofol anesthesia combined with INHA, incomplete data, age < 20 years; five cases were excluded (Fig 1).

No medication was administrated before anesthesia induction. Each patient received standard monitoring, including electrocardiography (lead II), noninvasive blood pressure testing, pulse oximetry, end-tidal carbon dioxide (EtCO_2_) measurement, central venous catheter insertion, and direct radial arterial blood pressure monitoring. Anesthesia was induced by fentanyl, propofol, and cisatracurium, or rocuronium in all patients [21].

As our previous reports [18–23], in brief, propofol anesthesia was maintained at an effect-site concentration (Ce) of 3.0–4.0 μg/mL by a target-controlled infusion (TCI) system (Fresenius Orchestra Primea; Fresenius Kabi AG, Bad Homburg, Germany); desflurane vaporizer was maintained between 4% and 10% (target minimum alveolar concentration of 0.7–1.3) [24]. During maintenance of anesthesia, all patients received FiO_2_ of 100% oxygen at a flow rate of 300 mL/min in a closed breathing system, and desflurane or Ce of propofol was adjusted downward and upward by 0.5–2% or Ce 0.2–0.5 μg/mL, respectively, if needed based on hemodynamics. Repetitive bolus injections of fentanyl and cisatracurium were administrated as necessary during surgery. The level of EtCO_2_ was maintained at 35–45 mmHg [18–23]. All patients were extubated and transferred to the intensive care unit after surgery. All patients received complete surgical resection as possible and perioperative steroid treatment with dexamethasone [15].

### Variables

We retrospectively gathered the following patient data: the type of anesthesia; time since the earliest included patient, which served as a surrogate of the calendar year; calendar period; sex; age at the time of surgery. We used the Charlson Comorbidity Index (CCI) to predict 10-year survival in patients with multiple comorbidities [21]. A Karnofsky performance status (KPS) score of ≤ 70 is a known poor prognostic factor; patients were grouped according to whether the score was 80–100 or ≤ 70 [25]. Preoperative functional capacity was assessed in metabolic equivalents (METs). As cardiac and long-term risks increase in patients with a functional capacity of < 4 METs during activities of daily living [26], patients were grouped according to whether the value was ≥ 4 METs or < 4 METs [21]. We also used the Clavien-Dindo classification, scaled from 0 (no complication) to V (most complications), to grade surgical complications. Other data included ASA physical status scores (ranging from I, indicating lowest morbidity, to V, indicating highest morbidity); tumor size; intraoperative blood transfusion; duration of surgery; duration of anesthesia; total opioid (fentanyl) use; postoperative radiation therapy; postoperative chemotherapy; the presence of postoperative recurrence. Because these variables have been shown or posited to affect patient outcomes, they were chosen as potential confounders [21].

### Statistical methods

The primary outcome was overall survival, which was compared between the propofol and desflurane anesthesia. The survival time was defined as the interval between the date of surgery and the date of death or March 02, 2020, for those who were censored. All data are shown as mean ± standard deviation (SD) or number (percentage) [21].

Mortality rates and patient characteristics were compared between the groups treated with the different anesthetics using Student’s *t* test or the chi-square test. The survival based on the type of anesthesia was depicted visually in a Kaplan-Meier survival curve. The association between the type of anesthesia (propofol or desflurane) and survival was analyzed by the Cox proportional-hazards model with and without adjustment for the abovementioned variables [21]. To avoid multi-collinearity, if there is a high correlation between the independent variables, it will be excluded in the multivariable analysis.

The propensity scores (PS) were created by simple logistic regression model in order to deal with the differences between propofol and desflurane groups. The model was build based on the abovementioned variables except“time since the earliest included patient” and “sex” due to lack of fit. We obtained 38 matched pairs based on one-to-one matching, using an R Package Matching (version 4.9–7) with calipers at 0.2 SD of the logit of the propensity score and without replacement. Propofol or desflurane anesthesia in a 1:1 ratio, to make sure the comparability between propofol and desflurane anesthesia before the surgery. Two-tailed *P*-values less than 0.05 were considered statistically significant.

## Results

The patient and treatment characteristics are shown in Table 1. There were more male patients in the desflurane (n = 36) than in the propofol anesthesia group (n = 25; *P* = 0.018). Time since the earliest included patient, calendar periods, age, CCI, KPS, preoperative functional status, ASA score, tumor size, intraoperative blood transfusion, duration of surgery and anesthesia, total fentanyl use, grade of surgical complications, use of postoperative radiotherapy, and use of postoperative chemotherapy showed insignificant differences between the two anesthetic techniques (Table 1).

**Table 1:** Patients’ and treatment characteristics and clinical outcomes for overall group and matched group after propensity scoring.

Variables	Overall Patients			Matched Patients		
	Propofol	Desflurane	*P* value	Propofol	Desflurane	*P* value
	(n = 53)	(n = 50)		(n = 38)	(n = 38)	
Time since the earliest included patient (years), Mean (SD)	5.2 (3.0)	4.8 (2.9)	0.504	5.6 (3.1)	4.0 (2.6)	0.022
Calendar period, n (%)			0.723			0.089
2008–2010	16 (30)	17 (34)		11 (29)	16 (42)	
2011–2013	16 (30)	17 (34)		10 (26)	14 (37)	
2014–2018	21 (40)	16 (32)		17 (45)	8 (21)	
Male sex, n (%)	25 (47)	36 (72)	0018	18 (47)	29 (76)	0.018
Age (years), Mean (SD)	57 (16)	58 (15)	0.787	58 (16)	57 (13)	0.957
Charlson comorbidityindex, Mean (SD)	4.5 (1.2)	4.5 (1.2)	0.969	4.6 (1.2)	4.4 (1.2)	0.575
Karnofsky performance status, Mean (SD)	88 (10)	87 (11)	0.488	88 (10)	88 (11)	1.000
≤ 70	9 (17)	13 (26)	0.381	7 (18)	8 (21)	1.000
80–100	44 (83)	37 (74)		31 (82)	30 (79)	
Functional status, n (%)			0.381			1.000
< 4MET	9 (17)	13 (26)		7 (18)	8 (21)	
≥ 4MET	44 (83)	37 (74)		31 (82)	30 (79)	
ASA, n (%)			0.381			1.000
II	44 (83)	37 (74)		31 (82)	30 (79)	
III	9 (17)	13 (26)		7 (18)	8 (21)	
Tumor size (cm), Mean (SD)	5.1 (1.3)	5.1 (1.3)	0.831	5.2 (1.4)	5.1 (1.4)	0.720
Intraoperative blood transfusion, n (%)	7 (13)	7 (14)	1.000	7 (18)	6 (16)	1.000
Duration of surgery (min), Mean (SD)	300 (32)	299 (32)	0.891	302 (33)	298 (31)	0.383
Duration of anesthesia (min), Mean (SD)	344 (36)	343 (36)	0.871	347 (37)	342 (35)	0.526
Total fentanyl use (μg). Mean (SD)	253 (58)	238 (66)	0.215	253 (60)	244 (65)	0.521
Grade of surgical complications, n (%)			0.494			0.209
0	45 (85)	46 (92)		33 (87)	36 (95)	
I	5 (9)	2 (4)		3 (8)	0 (0)	
II	3 (6)	2 (4)		2 (5)	2 (5)	
Postoperative radiotherapy, n (%)	37 (70)	32 (64)	0.677	29 (76)	25 (66)	0.448
Postoperative chemotherapy, n (%)	40 (76)	32 (64)	0.292	29 (76)	27 (71)	0.794
Postoperative recurrence, n (%)	44 (83)	48 (96)	0.070	31 (82)	36 (95)	0.156
All-cause mortality, n (%)	38 (72)	45 (90)	0.036	25 (66)	35 (92)	0.011
Cancer-specific mortality, n (%)	38 (72)	45 (90)	0.036	25 (66)	35 (92)	0.011

Data shown as mean ± SD or n (%). Grade of surgical complications: Clavien-Dindo classification.MET = metabolic equivalents; ASA = American Society of Anesthesiologists; N/A = not applicable.

The overall mortality rate or the cancer-specific mortality rate was significantly lower in the propofol anesthesia group (72.0%) than in the desflurane anesthesia group (90.0%) during follow-up (*P* = 0.036). The mean follow-up time was 2.5 years for the propofol group and 2.1 years for the desflurane group. Furthermore, the presence of postoperative recurrence did not differ between the two groups (Table 1).

**Fig 2:**
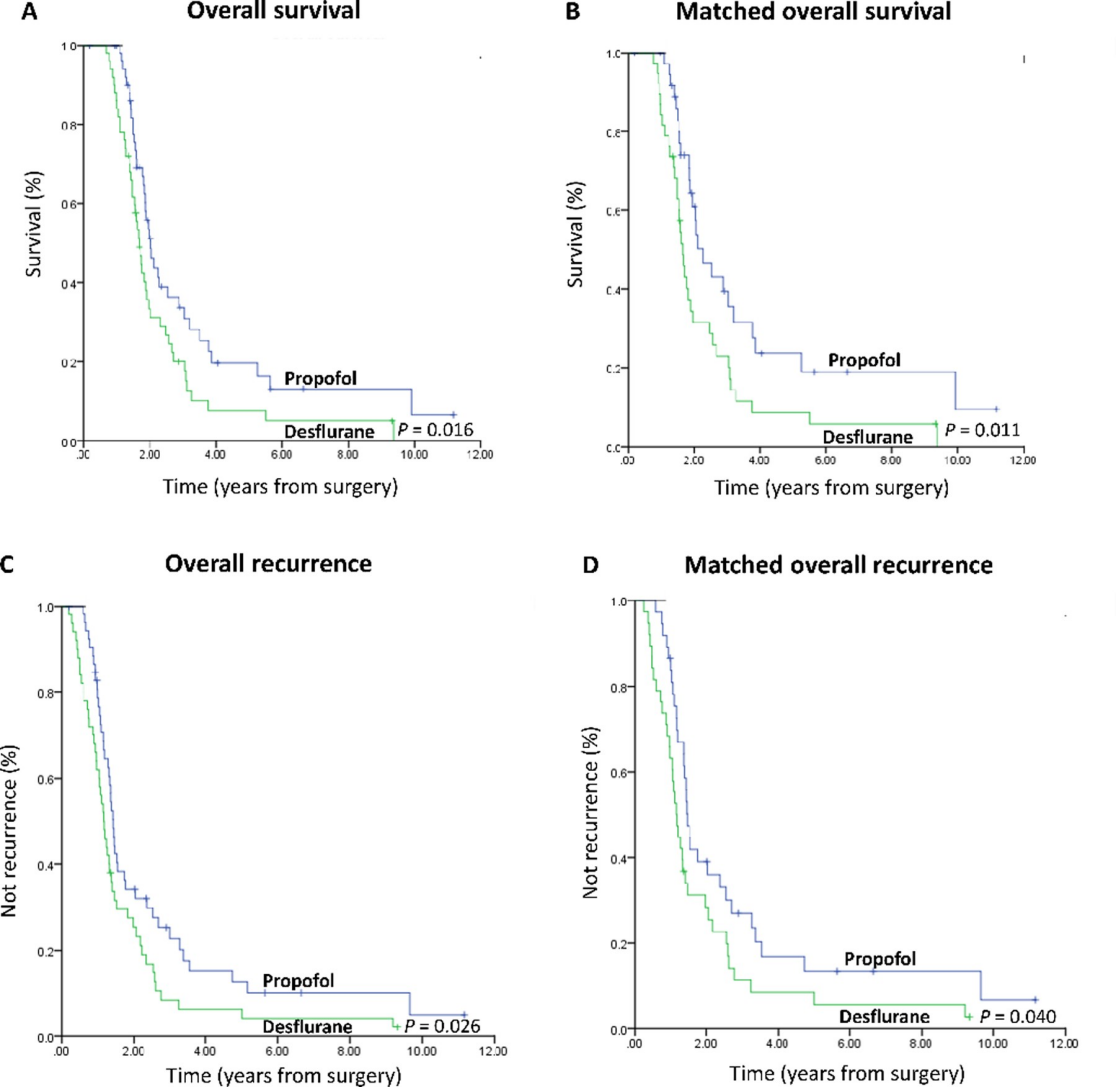
(A) Overall survival curves from the date of surgery by anesthesia type. (B) Overall survival curves from the date of surgery by anesthesia type after propensity score matching. (C) Overall recurrence curves from the date of surgery by anesthesia type. (D) Overall recurrence curves from the date of surgery by anesthesia type after propensity matching.

The overall mortality risk associated with propofol and desflurane use during GBM surgery is reported in Table 2. Overall survival from the date of surgery grouped according to the anesthetic technique and other variables was compared individually in a univariable Cox model and subsequently in a multivariable Cox regression model. Other variables that significantly increased the mortality risk were higher CCI, higher grade of surgical complications, and no postoperative radiotherapy after multivariable analysis (Table 2). KPS and functional status were excluded from the model due to they were the inverse of ASA. Recurrence was also excluded from the model since it is the intermediate variable. Patients with propofol anesthesia showed better overall survival than those with desflurane anesthesia (overall survival 40.0% versus 18.0%, respectively; the crude hazard ratio (HR) was 0.59 (95% confidence interval (CI), 0.38–0.91; *P* = 0.016). This finding did not change substantially in the multivariable analysis (HR, 0.48; 95% CI, 0.30–0.78; *P* = 0.003) (Table 2). Kaplan–Meier survival curves for the two anesthetic techniques are shown in Fig 2A.

**Table 2:** Cox proportional hazards regression for mortality: Univariable and multivariable models for overall patients.

	Univariable		Multivariable	
Variables	HR (95% CI)	*P* value	HR (95% CI)	*P* value
Anesthesia, Propofol (ref: Desflurane)	0.59 (0.38–0.91)	0.016	0.48 (0.30–0.78)	0.003
Time since the earliest included patient (years)	1.00 (0.92–1.10)	0.871		
Female (ref: Male)	0.94 (0.61–1.46)	0.780		
Age (years)	1.03 (1.01–1.05)	0.001	0.97 (0.92–1.02)	0.288
Charlson comorbidity index	1.46 (1.20–1.78)	<0.001	2.24 (1.05–4.80)	0.038
Karnofsky performance status, 80–100 (ref: ≤ 70)	0.43 (0.25–0.74)	0.002	N/A	
Functional status, ≥4 METs (ref: <4 METs)	0.43 (0.25–0.74)	0.002	N/A	
ASA, III, (ref: II)	2.32 (1.36–3.95)	0.002	0.63 (0.14–2.99)	0.565
Tumor size	0.99 (0.82–1.18)	0.876		
Intraoperative blood transfusion (ref: no)	0.87 (0.42–1.80)	0.702		
Duration of surgery (10 min)	0.98 (0.91–1.05)	0.525		
Duration of anesthesia (10 min)	0.98 (0.92–1.04)	0.505		
Total fentanyl use (10 μg)	0.94 (0.91–0.98)	0.001	0.98 (0.89–1.08)	0.723
Grade of surgical complications (ref: 0)				
I&II	2.77 (1.45–5.31)	0.002	3.83 (1.87–7.86)	<0.001
Postoperative radiotherapy (ref: no)	0.53 (0.34–0.85)	0.008	0.55 (0.32–0.95)	0.032
Postoperative chemotherapy (ref: no)	0.60 (0.38–0.96)	0.033	1.08 (0.61–1.89)	0.802
Postoperative recurrence (ref: no)	30.8 (2.39–398)	0.009	N/A	

Variables in the multivariable model were those significant variables in the univariable analyses, except Karnofsky performance status and functional status to avoid multi-collinearity. Since recurrence is the intermediate variable, it was excluded as well. MET = metabolic equivalents; ASA = American Society of Anesthesiologists; N/A = not applicable.

We used the PS from the logistic regression to adjust baseline characteristics and choice of therapy between the two anesthetic techniques due to significant differences in baseline characteristics between the two anesthetic techniques. Thirty-eight pairs were formed after matching (Table 1). Patient characteristics and prognostic factors of GBM showed insignificant differences between matched groups (except time since the earliest included patient and sex; Table 1). Kaplan-Meier survival curves for the two anesthetic techniques are shown in Fig 2B.

### Risk of postoperative recurrence, all-cause mortality, cancer-specific mortality by anesthesia type

Patients with propofol anesthesia had less postoperative recurrence than those with desflurane anesthesia; the crude HR was 0.63 (95% CI, 0.41–0.95; *P* = 0.026) (Fig 2C); the PS-matched HR was 0.60 (95% CI, 0.37–0.98; *P* = 0.040) (Fig 2D); the PS-matched HR with adjustment by time since the earliest included patient and sex was 0.53 (95% CI, 0.30–0.95; *P* = 0.034); and the PS-matched HR with adjustment by time since the earliest included patient, sex, surgeons, and anesthesiologists was 0.14 (95% CI, 0.03–0.68; *P* = 0.015) (Table 3).

**Table 3:** Risk of postoperative recurrence, all-cause mortality, cancer-specific mortality by anesthesia type.

Outcome Variables	Anesthesia	Crude-HR (95% CI)	*P* value	PS matched-HR (95% CI)	*P* value	PS matched-HR (Adjusted by time since the earliest included patient & sex; 95% CI)	*P* value	PS matched-HR (Adjusted by time since the earliest included patient & sex & surgeons & anesthesiologists; 95% CI)	*P* value
**Postoperative recurrence**	Desflurane	1.00		1.00		1.00		1.00	
	Propofol	0.63 (0.41–0.95)	0.026	0.60 (0.37–0.98)	0.040	0.53 (0.30–0.95)	0.034	0.14 (0.03–0.68)	0.015
**All-cause motality**	Desflurane	1.00		1.00		1.00		1.00	
	Propofol	0.59 (0.38–0.91)	0.016	0.51 (0.30–0.85)	0.011	0.45 (0.24–0.85)	0.014	0.15 (0.03–0.77)	0.023
**Cancer-specific mortality**	Desflurane	1.00		1.00		1.00		1.00	
	Propofol	0.59 (0.38–0.91)	0.016	0.51 (0.30–0.85)	0.011	0.45 (0.24–0.85)	0.014	0.15 (0.03–0.77)	0.023

HR = hazard ratio; PS = propensity score.

Analysis of all-cause mortality or cancer-specific mortality showed better survival in patients with propofol anesthesia than those with desflurane anesthesia. The crude HR was 0.59 (95% CI, 0.38–0.91; *P* = 0.016), and the PS-matched HR was 0.51 (95% CI, 0.30–0.85; *P* = 0.011); the PS-matched HR with adjustment by time since the earliest included patient and sex was 0.45 (95% CI, 0.24–0.85; *P* = 0.014); and the PS-matched HR with adjustment by time since the earliest included patient, sex, surgeons, and anesthesiologists was 0.15 (95% CI, 0.03–0.77; *P* = 0.023) (Table 3).

In summary, patients with desflurane anesthesia had higher all-cause mortality, higher cancer-specific mortality, and higher postoperative recurrence than those under propofol anesthesia. In addition, there was no occurrence of cardiovascular or adverse events in the two groups perioperatively.

## Discussion

A significant finding in the present study is that propofol anesthesia in GBM surgery is associated with better survival and a lower risk of postoperative recurrence than desflurane anesthesia. The result is consistent with our previous studies in which propofol anesthesia demonstrated better survival than desflurane anesthesia following cancer surgeries (intrahepatic cholangiocarcinoma, hepatocellular carcinoma, pancreatic cancer, gastric cancer, prostate cancer, and colon cancer) [18–23]. However, Sessler et al. [27] conducted a randomized controlled trial (RCT) among 2,108 women at 13 hospitals in Argentina, Austria, China, Germany, Ireland, New Zealand, Singapore, and the USA. They concluded that regional anesthesia-analgesia (paravertebral block and propofol) did not reduce breast cancer recurrence after minor curative surgery when compared to volatile anesthesia (sevoflurane) and opioids [27]. Enlund et al. [28] conducted an ongoing prospective, randomized, open-label, multicenter study on 8,000 patients who underwent radical surgery for breast or colorectal cancer. The primary outcome was 1-year and 5-year survival with propofol-based anesthesia compared with sevoflurane-based anesthesia. Dubowitz et al. [29] conducted a randomized, double-blind feasibility and pilot study of propofol-based anesthesia or volatile-based maintenance anesthesia during cancer resection surgery at three tertiary hospitals in Australia and the USA. This pilot study investigating anesthetic techniques and perioperative outcomes related to cancer shows feasibility for international and multicenter trials to provide evidence-based guidelines for the anesthetic management of patients undergoing major cancer surgery [29]. Therefore, we expect the two ongoing large RCTs [28, 29] to verify or refute that propofol anesthesia is better than volatile anesthesia for cancer surgery.

Surgical resection is the gold standard of therapy for solid and resectable tumors. However, surgery may suppress important host defenses and stimulate the development of recurrence. After the GBM surgery, the outcomes remain poor with a 5-year survival rate of 4–5%, and postoperative recurrence is nearly universal [2]. Postoperative recurrence has an impact on patient prognosis and survival in GBM. Thus, research on GBM has focused on developing strategies to ameliorate overall patient survival via reducing postoperative recurrence [30]. The plausibility of tumor recurrence depends on the balance between the cancer metastatic potential and the host defense, of which natural killer cell function and cell-mediated immunity are important parts [31]. Data from studies on human cancer cell lines and animal showed that different anesthetic techniques or anesthetics could influence immune response [4–9] and affect risks of cancer recurrence, metastasis, or patient survival [6, 8–11]. As INHA increased cerebral blood flow and intracranial pressure (ICP), which might threaten surgical exposure and postoperative neurofunction [32]. However, propofol was associated with improved ICP control and cerebral hemodynamics [33]. Therefore, propofol may improve prognosis in patients undergoing neurosurgery [17].

Grau et al. [15] showed that propofol anesthesia had no impact on patient survival when compared to INHA (isoflurane, desflurane, or sevoflurane) in GBM surgery. Schmoch et al. [16] reported that propofol anesthesia did not influence the survival of GBM patients compared with sevoflurane. Dong et al. [17] also showed that propofol anesthesia had no impact on cancer survival but reduced the risk of death in high-grade glioma patients with poor preoperative Karnofsky performance status (classification of functional impairment) compared with sevoflurane. To the best of our knowledge, no previous study has compared the effects of desflurane versus propofol anesthesia on patient outcomes after GBM surgery. Here, we found a 40% lower death rate with propofol anesthesia than desflurane anesthesia in GBM surgery. Our results suggest a potential effect in humans, although the magnitude of the observed effect is considerably larger than in previous studies. It seems biologically implausible that something as complicated as cancer can be reduced by more than a factor of two simply by anesthetic selection. Our results most likely overestimate the true treatment effect, which is common in retrospective studies. There are few studies on the influence of anesthetic techniques in GBM patients; further investigations are needed to examine the role of anesthetic techniques on postoperative recurrence in GBM surgery.

Data from human GBM cell lines support the influence of propofol on GBM cell growth and survival via different pathways [34–37]. Hsu et al. [34] reported that propofol activated reactive oxygen species-associated apoptosis involving human GBM cell cycle arrest. In addition, Xu et al. [35] showed that propofol could effectively suppress proliferation and invasion and induce the apoptosis of human GBM cells, at least partially through upregulation of microRNA-218 expression. Moreover, Liang et al. [36] found that propofol evoked Ca^2+^ movement and cell death in human GBM cells, though further clinical studies are needed. Xu et al. [37] reported that propofol inhibited Wnt signaling and exerted anticancer activity in glioma cells. However, Lai et al. [38] showed that sevoflurane promoted migration, invasion, and colony-forming ability of human GBM cells by possibly increasing cell surface protein 44 expression. Besides, Zhang et al. [39] demonstrated that sevoflurane suppressed migration and invasion of glioma cells by regulating microRNA-146b-5p and matrix metallopeptidase-16. However, there is no study in the literature on the effect of desflurane on glioma cells. Thus, propofol may reduce GBM tumor growth, thus decreasing the risk of recurrence, whereas INHA may cause opposite effects on GBM tumor growth.

Upregulation of hypoxia-inducible factor (HIF) was associated with a poor prognosis in one clinical cancer study [40]. Reports suggested that propofol reduced HIF-1α expression in prostate cancer and non-small-cell lung cancer cell lines [41, 42]. Moreover, a recent study showed that propofol could protect against hypoxia-mediated impairment of blood-brain barrier integrity because HIF-1α expression was increased by hypoxia and alleviated by propofol [43]. In contrast, volatile anesthetics enhanced HIF expression [41, 44]. Meanwhile, HIF-1α was overexpressed in GBM [45], and a knockdown of HIF-1α suppressed the migration and invasion of GBM cells [46]. Together, these limited reports suggest that the administration of INHA may stimulate HIF-1α expression, whereas propofol has a beneficial effect by suppressing HIF-1α expression.

This study also found that a higher CCI score, a higher grade of surgical complications, and no postoperative radiotherapy were associated with poor survival after GBM surgery, as has been observed previously [47–49]. Further investigation is still necessary.

There were some limitations in this study. First, it was retrospective, and the 103 patients were not randomly allocated. However, we used all available patients from January 2008 to December 2018 from the medical center. Patient characteristics such as sex differed significantly between the groups, and we conducted PS matching to address this issue. But the model was based on the abovementioned variables such as age, CCI, KPS, functional status, ASA score, and tumor size, except “time since the earliest included patient” and “sex” due to lack of fit. However, the findings did not change substantially using further adjustment by time since the earliest included patient and sex (Table 3). Second, we analyzed only GBM because it is the most common malignant primary brain tumor [1]. Third, different volatile anesthetics may have varying effects on GBM. This study focused on desflurane because it is the most frequently used INHA in our hospital. Fourth, a previous study reported that high-volume surgeons were significantly associated with positive patient outcomes in brain tumor resection [50]. Moreover, the anesthesiologists chose the type of anesthesia, which may have been subject to original selection bias between propofol and INHA. Therefore, we conducted PS matched-HR with further adjustment by surgeons and anesthesiologists, and these factors did not affect the outcome (Table 3); further investigation is needed for surgeon or anesthesiologist volume in GBM patient outcomes. Finally, patients maintained with desflurane also received single bolus 1–2 mg/kg propofol for induction of anesthesia, and its effect on our findings is unknown [17]. However, Schaefer et al [51]. reported that the increasing doses of propofol (per 10 mg/kg) did not associate with decreased one-year mortality in patients with solid tumors.

In conclusion, during GBM surgery, propofol anesthesia was associated with better survival than desflurane anesthesia. Further, patients under desflurane anesthesia exhibited more postoperative recurrence.

## Supporting information

S1 Data (XLSX)

## List of abbreviations

ASA: American Society of Anesthesiology
Ce: effect-site concentration
CCI: Charlson comorbidity index
CI: confidence interval
EtCO_2_: end-tidal carbon dioxide
GBM: glioblastoma
HR: hazard ratio
HIF: hypoxia-inducible factor
IRB: institutional review board
INHA: inhalation anesthesia
ICP: intracranial pressure
KPS: Karnofsky performance status
METs: metabolic equivalents
PS: propensity score
RCT: randomised controlled trial
SD: standard deviation
TSGH: Tri-Service General Hospital
TCI: target controlled infusion
