# Supplementary material for: Propofol-based total intravenous anesthesia is associated with better survival than desflurane anesthesia in glioblastoma surgery
Source: PLoS One. 2021 Aug 5;16(8):e0255627. doi: 10.1371/journal.pone.0255627 (PMC8341516; doi:10.1371/journal.pone.0255627)
Supplement: Attachment — Submitted filename: Reply_template_PONE_R1.doc [file pone.0255627.s002.doc]

**Reply to the reviewers’ comments**

| Reviewer Number | Original comments of the reviewer | Reply by the author(s) | Changes done on page number and line number |
| --- | --- | --- | --- |
| 1 | 1. All patients received a small propofol dose for induction. Do the Authors believe that this small dose might have had any influence? | Thanks for your kindly reminder. However, in the recent study, this small dose (<10 mg/kg) might not affect solid cancer survival. ( Br J Anaesth 2020;124:271-280.) | P21, Line 4-7. |
| 1 | 2. Another major issue with volatile anesthetics vs propofol is the potential cardioprotective effect. Both anesthetics have been suggested to have cardioprotective effects, with controversial results. Do the Authors have any data on cardiovascular vs cancer-related mortality/adverse events? This would add another interesting piece of information. | Thanks for your kindly reminder. We have traced the medical records again, but there is no occurrence of  cardiovascular or adverse events in two groups perioperatively. | P12, Line 4-5. |
| 1 | 3. Which was the target MAC for the desflurane group? | The target MAC of 0.7–1.3 in the desflurane. (N Engl J Med. 2011;365:591-600.) | P11, Line 20. |
| 1 | 4. It is unclear what does "time since the earliest included patient" mean? | Time since the earliest included patient, which served as a surrogate of the calendar year. | P12, Line 7-8. |
| 1 | 5. Do the Authors have data on duration of surgery/anesthesia? | Thanks for your kindly reminder, we add data on duration of surgery/anesthesia in Table 1 and 2. Finally, duration of surgery/anesthesia was insignificantly different between the two groups. | Table 1 and 2. |
| 1 | 6. Please discuss the following ongoing large RCTs on the topic:  - Enlund M, Enlund A, Berglund A, Bergkvist L. Rationale and Design of the CAN Study: an RCT of Survival after Propofol- or Sevoflurane-based Anesthesia for Cancer Surgery. Curr Pharm Des. 2019;25(28):3028-3033. doi:10.2174/1381612825666190705184218  - Dubowitz JA, Cata JP, De Silva AP, et al. Volatile anaesthesia and peri‐operative outcomes related to cancer: a feasibility and pilot study for a large randomised control trial. Anaesthesia. January 2021. doi:10.1111/anae.15354  - Sessler DI, Pei L, Huang Y, et al. Recurrence of breast cancer after regional or general anaesthesia: a randomised controlled trial. Lancet. 2019;394(10211):1807-1815. doi:10.1016/S0140-6736(19)32313-X. | Thanks for your kindly reminder, we revised the manuscript as you suggested. Sessler et al. conducted a randomized controlled trial (RCT) among 2,108 women at 13 hospitals in Argentina, Austria, China, Germany, Ireland, New Zealand, Singapore, and the USA. They concluded that regional anesthesia-analgesia (paravertebral block and propofol) did not reduce breast cancer recurrence after minor curative surgery when compared to volatile anesthesia (sevoflurane) and opioids. ( Lancet. 2019;394:1807-1815.) Enlund et al. conducted an ongoing prospective, randomized, open-label, multicenter study on 8,000 patients who underwent radical surgery for breast or colorectal cancer. The primary outcome was 1-year and 5-year survival with propofol-based anesthesia compared with sevoflurane-based anesthesia. (Curr Pharm Des. 2019;25:3028-3033.) Dubowitz et al. conducted a randomized, double-blind feasibility and pilot study of propofol-based anesthesia or volatile-based maintenance anesthesia during cancer resection surgery at three tertiary hospitals in Australia and the USA. This pilot study investigating anesthetic techniques and perioperative outcomes related to cancer shows feasibility for international and multicenter trials to provide evidence-based guidelines for the anesthetic management of patients undergoing major cancer surgery. (Anaesthesia. January 2021. doi:10.1111/anae.15354) Therefore, we expect the two ongoing large RCTs to verify or refute that propofol anesthesia is better than volatile anesthesia for cancer surgery. | P17, Line 7-21. |
| 2 | 1. The article is understandable in its present form, but requires some improvement of the English language. I suggest a thorough review of grammar with a native speaker. | Thanks for your kindly reminder, we revised the manuscript as you suggested (please see English-editing certification below). | Reply to the reviewers’ comments P4 and the “Supporting Information file”. |
| 2 | 2. Were all surgical resections considered complete or where these cases of incomplete resection? If not, an estimate of the extent of resection may be a worthwhile addition. | In fact, all patients received complete surgical resections as possible. | P12, line 3-4. |
| 2 | 3. It might be of interest to include total opioids used if retrievable, as it may differ between the two types of anesthesia and may have an impact on the immune system as well. | Thanks for your suggestion, we add data on total opioids use in Table 1 and 2. Finally, total fentanyl use was insignificantly different between the two groups. | Table 1 and 2. |
| 2 | 4. I suggest more information on the preoperative status of these patients, such as the Karnofsky performance status, as it may affect outcome. METs allow us to infer some of this information, but offer only two very broad categories as used in the manuscript. | Thanks for your suggestion, we add data on Karnofsky performance status in Table 1 and 2. Finally, Karnofsky performance status was insignificantly different between the two groups. | Table 1 and 2. |
