# Supplementary material for: Propofol-based total intravenous anesthesia is associated with better survival than desflurane anesthesia in glioblastoma surgery
Source: PLoS One. 2021 Aug 5;16(8):e0255627. doi: 10.1371/journal.pone.0255627 (PMC8341516; doi:10.1371/journal.pone.0255627)
Supplement: Attachment — Submitted filename: Reply_template_PONE_R2.doc [file pone.0255627.s003.doc]

**Reply to the reviewers’ comments**

| Reviewer Number | Original comments of the reviewer | Reply by the author(s) | Changes done on page number and line number |
| --- | --- | --- | --- |
| 1 | 1. I believe that all of my comments have been adequately addressed. Please move the description of adverse cardiovascular events to the Results rather than the Methods section | Thanks for your kindly reminder. We have moved the description of adverse cardiovascular events from the Methods section to Results section. | P16, Line 5-6. |
| 2 | 1. As an additional point, I would suggest implementing the definition of the criteria used for multi-collinerarity in the methods section and a brief description of the reasons for exclusion in the results section, to better understand the exclusion of Karnofsky performance status, functional status and recurrence from the final multivariate model. | Thank you for the suggestions. The points are well taken. We have added relevant descriptions in the Methods, Results as well as Footnote of Table 2. | 1. P13, Line 7-9. 2. P14, Line 19-20. 3. P31, Footnote of Table 2. |
|  | We have to revise Dr. Zhi-Fu Wu’s affiliations as following: Zhi-Fu Wu, MD^1,2,3^ | Name of Department(s) and Institution(s):  1. Department of Anesthesiology, Tri-Service General Hospital and National Defense Medical Center, Taipei, Taiwan, Republic of China.  2. Department of Anesthesiology, Kaohsiung Medical University Hospital, Kaohsiung Medical University, Kaohsiung, Taiwan, Republic of China.  3. Department of Anesthesiology, Faculty of Medicine, College of Medicine, Kaohsiung Medical University, Kaohsiung, Taiwan, Republic of China. | 1. P1, Line 3.  2. P1, Line 10-11.  3. P2, Line 11-13. |
